# *SOX10 *directly modulates *ERBB3 *transcription via an intronic neural crest enhancer

**DOI:** 10.1186/1471-213X-11-40

**Published:** 2011-06-14

**Authors:** Megana K Prasad, Xylena Reed, David U Gorkin, Julia C Cronin, Anthony R McAdow, Kristopher Chain, Chani J Hodonsky, Erin A Jones, John Svaren, Anthony Antonellis, Stephen L Johnson, Stacie K Loftus, William J Pavan, Andrew S McCallion

**Affiliations:** 1 McKusick-Nathans Institute of Genetic Medicine, The Johns Hopkins University School of Medicine, Baltimore, Maryland 21205, USA; 2 Genetic Disease Research Branch, National Human Genome Research Institute, National Institutes of Health, Bethesda, Maryland 20892, USA; 3 Department of Genetics, Washington University School of Medicine, St. Louis, Missouri 63110, USA; 4 Department of Human Genetics, University of Michigan Medical School, Ann Arbor, Michigan 48109, USA; 5 Department of Neurology, University of Michigan Medical School, Ann Arbor, Michigan 48109, USA; 6 Program in Cellular and Molecular Biology; University of Wisconsin-Madison, Madison, Wisconsin 53705, USA; 7 Department of Comparative Biosciences, University of Wisconsin-Madison, Madison, Wisconsin 53705, USA; 8 Department of Molecular and Comparative Pathobiology, The Johns Hopkins University School of Medicine, Baltimore, Maryland 21205, USA

## Abstract

**Background:**

The *ERBB3 *gene is essential for the proper development of the neural crest (NC) and its derivative populations such as Schwann cells. As with all cell fate decisions, transcriptional regulatory control plays a significant role in the progressive restriction and specification of NC derived lineages during development. However, little is known about the sequences mediating transcriptional regulation of *ERBB3 *or the factors that bind them.

**Results:**

In this study we identified three transcriptional enhancers at the *ERBB3 *locus and evaluated their regulatory potential *in vitro *in NC-derived cell types and *in vivo *in transgenic zebrafish. One enhancer, termed *ERBB3*_MCS6, which lies within the first intron of *ERBB3*, directs the highest reporter expression *in vitro *and also demonstrates epigenetic marks consistent with enhancer activity. We identify a consensus SOX10 binding site within *ERBB3*_MCS6 and demonstrate, *in vitro*, its necessity and sufficiency for the activity of this enhancer. Additionally, we demonstrate that transcription from the endogenous *Erbb3 *locus is dependent on Sox10. Further we demonstrate *in vitro *that Sox10 physically interacts with that *ERBB3*_MCS6. Consistent with its *in vitro *activity, we also show that *ERBB3*_MCS6 drives reporter expression in NC cells and a subset of its derivative lineages *in vivo *in zebrafish in a manner consistent with *erbb3b *expression. We also demonstrate, using morpholino analysis, that Sox10 is necessary for *ERBB3*_MCS6 expression *in vivo *in zebrafish.

**Conclusions:**

Taken collectively, our data suggest that *ERBB3 *may be directly regulated by SOX10, and that this control may in part be facilitated by *ERBB3*_MCS6.

## Background

The neural crest (NC) is a transient, multipotent and migratory population of cells present in early vertebrate development. NC cells arise from the lateral folds of the neural plate at neurulation and give rise to a multitude of cell types including pigment cells, neurons and glia of the peripheral nervous system, craniofacial skeleton and cartilage, and adrenal medullary cells [1]. Defects in NC development underlie several human diseases such as Waardenburg syndrome, Hirschsrpung disease and DiGeorge syndrome, which present a spectrum of phenotypes including craniofacial defects, ocular, pigmentary and otic defects, enteric hypoganglionosis, and cardiac malformations [2,3]. Despite significant recent progress in the identification of key signaling pathways and transcription factors involved in NC induction, the hierarchical relationships between these pathways and factors are not well understood.

One critical gene in this network is *Erbb3*, a receptor tyrosine kinase that belongs to the epidermal growth factor (EGF) receptor family. Other members of the family include receptors such as *Egfr1*, *Erbb2 *and *Erbb4 *[4]. The structure of Erbb3 includes an extracellular domain, which interacts with ligands Neuregulin1 and Neuregulin2, and a cytoplasmic domain. Unlike other members of the EGF receptor family, the cytoplasmic domain of Erbb3 lacks tyrosine kinase activity [5,6]. Erbb3 is therefore thought to heterodimerize with Erbb2, which lacks a cognate receptor but possesses tyrosine kinase activity, in order to activate downstream pathways [7,8]. Upon activation, Erbb3 triggers several downstream pathways such as PI3K, MAPK, protein kinase C, Jak-STAT and PLCγ [4].

*Erbb3 *plays important roles in the development of the NC and its derivative tissues. Consistent with these observations, expression analyses in mouse and zebrafish have detected *Erbb3*/*erbb3b *transcripts in pre-migratory and migratory NC, and various NC derivatives including, cranial ganglia, posterior lateral line ganglia and Schwann cells [9-14]. Expression has also been observed in non-NC derived populations including the brain, olfactory lobes and myotome [10,14]. Furthermore, mice harbouring targeted mutant alleles of *Erbb3 *exhibit defects in the formation of NC and its derivatives. Homozygous null mice display a dramatic reduction in numbers of Schwann cells, hypoplastic cardiac cushion mesenchyme and cardiac valves, defects in cranial ganglia formation and cerebellar hypoplasia [9,13]. *Erbb3 *deficient mice also demonstrate a lack of chromaffin cells in the adrenal medulla [9]. Furthermore, targeted deletions of *Nrg1 *and *Erbb2*, a ligand and a binding partner of Erbb3 respectively, result in NC cell migration defects [15]. More recently, several *in vitro *and zebrafish studies have revealed a potential role for *Erbb3 *in melanocyte development and in melanoma [16-18]. Consistent with these data, *Erbb3 *has been implicated in other neoplasias including breast cancer and lung cancer [19]. However, what factors act upstream of *Erbb3 *to direct its expression and function in the variety of cell types derived from NC cells is not well understood. Even less well understood is the genomic sequence basis of the transcriptional regulatory control that facilitates the cell fate decisions and homeostatic maintenance of these cells during development

One factor proposed to regulate *Erbb3 *is *Sox10*, a member of the SoxE family of transcription factors [20]. *Sox10 *is indispensible for proper neural crest development and loss of *Sox10 *leads to reductions of specific NC derivatives including peripheral neurons and melanocytes [21,22]. Also, *SOX10 *mutations have been discovered in several patients with Waardenburg syndrome and syndromic Hirschsprung disease [23]. However, data generated to date linking *Sox10 *and *Erbb3 *have been largely correlative. Both *Sox10 *and *Erbb3 *share overlapping expressions patterns and deletions of *Sox10 *and *Erbb3 *affect overlapping cell populations such as Schwann cells, cranial ganglia and sympathetic neurons. In mice where the endogenous *Sox10 *locus has been replaced by a LacZ cassette, *Erbb3 *expression is initiated in premigratory NC but is lost once the cells begin migrating [10], suggesting *Erbb3 *regulation by *Sox10*. Furthermore *Erbb3 *transcript levels increase upon overexpression of *Sox10 *in Neuro2A neuroblastoma cells [10]. However, it is not known if this regulation is direct or indirect because the regulatory topography of *Erbb3 *has not yet been uncovered, and nor have any *Sox10 *responsive elements been demonstrated at the *Erbb3 *locus.

In this study, we address the transcriptional regulation of *ERBB3 *during neural crest development. We use sequence constraint as a metric predictive of function to identify putative regulatory elements at the human *ERBB3 *locus. We test the regulatory potential of these elements *in vitro *in two cell lines representative of neural crest derived tissues. We also test each element for epigenetic marks consistent with enhancer activity. We verify enhancer activity of each element *in vivo *using zebrafish transgenesis, identifying three transcriptional enhancers of *ERBB3*. One intronic enhancer termed *ERBB3*_MCS6 displays the strongest enhancer activity *in vitro *and high enrichment for H3K4me1. We demonstrate that this enhancer is responsive to and dependent on *Sox10 *for its regulatory behaviour. Similarly, we demonstrate that Sox10 is necessary and sufficient for activation of the endogenous *Erbb3*. Furthermore, we also demonstrate that *ERBB3*_MCS6 directs expression in NC cells and their derivative lineages *in vivo *in zebrafish, and that the requirement for Sox10 for its regulation is maintained *in vivo *as well. Therefore, we conclude that *SOX10 *likely contributes to the transcriptional modulation of *ERBB3 *acting at least in part directly via *ERBB3*_MCS6.

## Results

### Multi-species conserved sequences (MCS) at the *ERBB3 *locus direct reporter expression in *Erbb3 *expressing cell lines and show epigenetic marks consistent with enhancer activity

**Figure 1:**
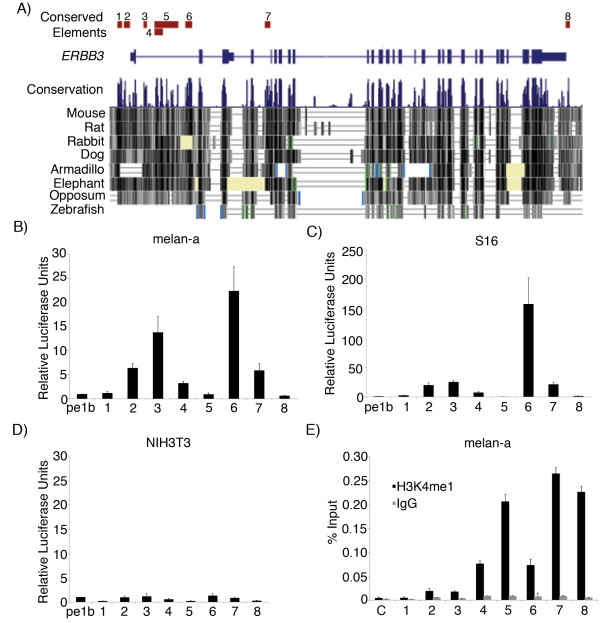
**Multi-species conserved sequences at the *ERBB3 *drive reporter activity in neural crest derived lines and exhibit epigenetic marks consistent with enhancer activity**. (A) Schematic representation of the eight multi-species conserved sequences at the human *ERBB3 *locus. (B) Luciferase activity of *ERBB3*_MCS1-8 in melan-a cells. (C) Luciferase activity of *ERBB3*_MCS1-8 in S16 cells. (D) Luciferase activity of *ERBB3_*MCS1-8 in NIH3T3 cells. All luciferase values are normalized to internal renilla control and are shown as fold-change in reporter activity as compared to the promoter only construct (pe1b) with standard deviation. (E) Real-time PCR results of Chromatin Immunoprecipitation. Black bar indicates enrichment upon H3K4me1 ChIP whereas grey bar indicates enrichment with non-specific IgG. Error bars indicate standard deviation of technical replicates in real-time PCR. Elements MCS1-8 represent the mouse orthologs of ERBB3_MCS elements. C refers to a region 3 Mb upstream of *Erbb3*, which was used as a negative control.

As in previous studies, we used sequence constraint to identify putative regulatory enhancers of *ERBB3 *[24,25]. Briefly, we identified evolutionarily conserved non-coding sequences at the human *ERBB3 *locus using Phastcons (http://genome.ucsc.edu/) [26], selecting eight sequences for functional analyses (LOD 30-196; Methods; Figure 1A). We amplified and cloned each of the sequences upstream of an E1B minimal promoter driving a luciferase reporter. We tested the regulatory potential of each of the sequences to drive transcription via luciferase assays in two NC derived cell lines, cultured mouse melanocytes (melan-a) and cultured rat Schwann cells (S16), both of which express *Erbb3 *(Figure 1B, C). Of the eight MCS elements amplified and tested, five (*ERBB3*_MCS2, MCS3, MCS4, MCS6 and MCS7) directed luciferase reporter expression at levels significantly higher than the promoter only luciferase construct in both cell lines (p≤0.003) (Figure 1B, C). Furthermore, *ERBB3*_MCS6 showed the greatest increase in luciferase activity- approximately 23-fold greater than the promoter only construct (p = 0.002) in melan-a cells and approximately 150-fold greater than the promoter only construct in S16 cells. Interestingly, although *ERBB3*_MCS5 overlaps *ERBB3*_MCS4, it demonstrates lower luciferase activity than *MCS4 *in melan-a cells. This suggests that there may be transcriptional repressor elements in *ERBB3*_MCS5 that are causing it to drive lower reporter expression in the luciferase assay. When the eight *ERBB3*_MCS elements were tested in a cell line that does not express *Erbb3 *(NIH3T3), none directed higher luciferase activity than the promoter only construct (Figure 1D). Therefore, this suggests that *ERBB3*_MCS 2, MCS3, MCS4, MCS6 and MCS7 are potentially enhancers of *ERBB3 *expression in the NC.

In order to determine whether these MCS elements were indeed enhancers within their genomic context, we assayed the presence of an epigenetic mark previously shown to be predictive of enhancer function. Since H3K4 monomethylation (H3K4me1) has been shown to be enriched at transcriptional enhancers, we used chromatin immunoprecipitation (ChIP) to determine whether H3K4me1 marks were enriched at the mouse orthologs of *ERBB3_*MCS elements within the genomic context of melan-a cells [27]. We used an antibody against H3K4me1 and an isotype control IgG to immunoprecipitate sheared crosslinked chromatin from these cells. We analyzed immunoprecipitation using real-time PCR and calculated enrichment using the percent input method (Figure 1E). *ERBB3*_MCS1, MCS2 and MCS3 showed consistently low levels of enrichment upon immunoprecipitation with the H3K4me1 antibody as compared to the IgG control samples, whereas *ERBB3*_MCS4, MCS5, MCS6, MCS7 and MCS8 showed consistently high levels of enrichment upon immunoprecipitation as compared to the IgG samples. A control sequence 3 Mb upstream of *ERBB3 *was not enriched in H3K4me1 samples as compared to IgG samples. *ERBB3*_MCS5, MCS7 and MCS8 showed the highest levels of enrichment as compared to the IgG samples. This contrasts with the low luciferase activity driven by these sequences *in vitro*, consistent with reports suggesting that H3K4me1 is not sufficient to distinguish between functionally active and inactive enhancers [28,29]. Elements *ERBB3*_MCS2, MCS3, MCS4 and MCS6 however did show increased luciferase activity as well as H3K4me1 marks, suggesting that these sequences may be functional endogenous enhancers of *ERBB3 *in NC-derived cell types.

### *ERBB3*_MCS6 harbours putative transcription factor binding sites for key neural crest transcription factors

Since *ERBB3*_MCS6 demonstrated the highest luciferase activity *in vitro *and showed enrichment for H3K4me1 consistent with a role for *ERBB3*_MCS6 as an enhancer of *ERBB3*, we focused our subsequent analyses on this element. To determine what pathways or transcription factors may be acting upon this element and hence potentially modulating *Erbb3 *expression, we queried three publicly available transcription factor identification databases (JASPAR, MATCH and TRANSFAC) to identify putative transcription factor binding sites (TFBSs) within this sequence. To refine our search further we prioritized sites that were found by two or more TF databases, selecting those that corresponded to transcription factors with known roles in NC development (SOXE and AP2). We identified two SOXE sites (SOXE-1 and SOXE-2) and one AP2 site (Additional file 1, Figure S1). Members of the SOXE family, SOX8, SOX9 and SOX10, bind to the SOXE consensus site 5'-AACAAT-3'. The identification of SOXE family (SOX8, SOX9 and SOX10) motifs is consistent with the predicted role for *Sox10 *in *Erbb3 *modulation. SOXE proteins typically bind the consensus site 5'-AACAAT-3'. Both SOXE-1 and SOXE-2 contain the 5'-ACAAT-3' core sequence but differ from the consensus in that the 5'-most adenine is replaced by a cytosine. *AP2 *is a retinoic acid inducible transcription factor that binds to the consensus sequence 5'-CCCCAGGC-3' [30,31]. It is expressed in the NC and its derivatives including cranial and sensory ganglia and facial mesenchyme and has a known role in the regulation of *ERBB3 *expression [32-36]. The *ERBB3 *promoter harbours potential AP2 binding sites, causing speculation of the role of AP2 as an *ERBB3 *regulator [37].

### SOXE2 is required for enhancer activity of *ERBB3*_MCS6

**Figure 2:**
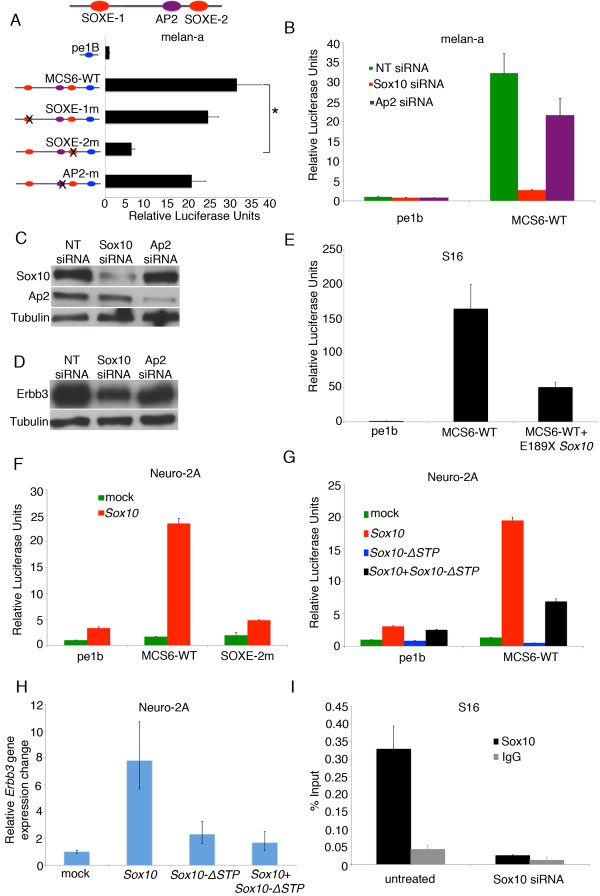
**Sox10 is necessary and sufficient for the activity of *ERBB3*_MCS6 *in vitro***. (A) Luciferase activities of wild-type *ERBB3_*MCS6 and TFBS mutations in *ERBB3_*MCS6 in melan-A cell line. The position of each TFBS in *ERBB3_*MCS6 is shown on top and the corresponding mutation in each TFBS is shown next to the luciferase value for that construct. * indicates statistical significance. (B) Luciferase activity of WT *ERBB3_*MCS6 in melan-A cells in mock-transfected cells and in cells with transient *Sox10 *and *Ap2 *knockdown. (C) Western blot to confirm knockdown of Sox10 and Ap2 protein upon siRNA treatment. Tubulin antibody was used as a loading control. (D) Western blot showing Erbb3 protein levels in melan-a cells upon transient transfection with *Sox10 *and *Ap2 *siRNA or mock-transfected cells. Tubulin antibody was used as a loading control. (E) Luciferase activity of *ERBB3*_MCS6 upon knockdown of Sox10 in S16 cells using a dominant negative *SOX10 *mutant (E189X) under a CMV prmoter. (F) Luciferase assay of WT and SOXE-2m *ERBB3_*MCS6 in Neuro2A cells when transiently co-transfected with an empty expression vector (pcDNA.31) and *Sox10 *cDNA. Cell lysates were collected 24 hours post transfection. (G) Luciferase assay of WT *ERBB3_*MCS6 in Neuro2A cells when transiently co-transfected with equal amounts of WT and *Sox10-ΔSTP *cDNA either individually or in combination. Cell lysates were collected 24 hours post transfection. All luciferase values are normalized to renilla internal control and shown as fold-change compared to promoter only construct (pe1B) with standard deviation. (H) Real-time PCR of *Erbb3 *transcript levels upon expression of WT and *Sox10-ΔSTP *cDNA individually and in combination. Values are normalized to 18s internal control and are shown as fold-change compared to promoter only construct (pcDNA3.1) with standard error. (I) Real-time PCR of ChIP against Sox10 in untreated S16 cells and in S16 cells treated with Sox10 morpholino. Black bar indicates enrichment upon Sox10 ChIP whereas grey bar indicates enrichment with non-specific IgG. Error bars indicate standard deviation of technical replicates in real-time PCR.

We set out to determine the importance of the SOXE and AP2 transcription factor families in modulating the activity of *ERBB3*_MCS6 by mutating predicted binding sites in the context of a pe1b promoter driving a luciferase reporter. We then compared the luciferase activity of each mutant construct to that of the wild-type *ERBB3*_MCS6 sequence in the melan-a cell line, which expresses *Sox10*, *Erbb3 *and *Ap2 *(Figure 2A). Mutation of the SOXE2 but not the SOXE1 motif significantly reduced enhancer activity of *ERBB3*_MCS6 (Figure 2A, p = 0.002 for SOXE2). This observation is consistent with a suggested role for *Sox10 *in regulating *Erbb3 *in NC-derived populations [10]. However, since mutation of the SOXE-2 site does not completely abrogate *ERBB3*_MCS6, this suggests that additional factors may also be responsible for the activity of *ERBB3*_MCS6. By contrast, mutation of the AP2 site does not significantly reduce luciferase activity of *ERBB3*_MCS6 across replicate experiments. However, we cannot exclude the presence of a non-canonical AP2 site that may be required for *ERBB3*_MCS6 activity independent of or in addition to this site.

### Sox10 is necessary for *ERBB3*_MCS6 enhancer activity and *Erbb3 *expression

Since mutation of the SOXE-2 binding site caused a significant reduction in *ERBB3*_MCS6 enhancer activity, we asked whether *Sox10 *may be acting through *ERBB3_*MCS6 to regulate *Erbb3 *in NC populations. We used siRNA (SMARTpool, Dharmacon) to transiently knock down *Sox10 *gene product in melan-a cells and assayed the effect of the knockdown on *ERBB3*_MCS6 activity via luciferase assays, as before. Consistent with significantly reduced *ERBB3_*MCS6 activity (Figure 2B; p = 0.05). Sox10 knockdown was confirmed via Western blot as shown in Figure 2C. We also determined the effect of *Sox10 *knockdown on endogenous Erbb3 protein levels in melan-a cells. As shown in figure 2D, knockdown of *Sox10 *causes a decrease of endogenous Erbb3 protein levels. Similarly, we interfered with Sox10 protein function in S16 cells using a previously characterized construct expressing a dominant negative mutant form of Sox10 (E189X) under the control of a CMV promoter [38]. The E189X mutant protein has an intact DNA binding domain but lacks a transactivation domain. Upon transfection of S16 cells with this mutant cDNA of *Sox10*, there was a significant decrease in luciferase activity of *ERBB3*_MCS6 (Figure 2E). Taken together, these experiments demonstrate that *Sox10 *is necessary for the expression of *Erbb3 *and for enhancer activity of *ERBB3_MCS6*, consistent with its predicted role in modulating *Erbb3*. Knockdown of *Ap2 *in melan-a cells also led to a significant reduction in *ERBB3_*MCS6 transcriptional activity (Figure 2B). Furthermore, *Ap2 *knockdown also reduced endogenous Erbb3 protein levels (Figure 2D), consistent with previous evidence for a role for *AP2 *in regulating *ERBB3 *[35,36].

### *Sox10 *is sufficient for *ERBB3*_MCS6 enhancer activity and *Erbb3 *expression

Given that Sox10 is necessary for *ERBB3*_MCS6 enhancer activity, we next tested whether *Sox10 *is also sufficient for *ERBB3_*MCS6 activity. We transiently co-transfected mouse neuroblastoma cells (Neuro2A) with the *ERBB3_*MCS6 luciferase vector and with either a construct expressing wild-type *Sox10 *cDNA under the control of a CMV promoter (pcDNA3.1) or with an empty expression vector. Neuro2A cells were selected due to low expression levels of *Sox10 *and *Erbb3 *and because this cell line has been previously used to study the interaction between *Sox10 *and *Erbb3 *[10]. In the presence of *Sox10*, the transcriptional activity of *ERBB3_*MCS6 increased almost 25-fold, compared to cells co-transfected with the empty vector (Figure 2F). In the absence of *Sox10*, *ERBB3_*MCS6 does not display significant transcriptional activity in Neuro2A cells. Further, consistent with a role for *Sox10 *in directly regulating MCS6, mutation of the SOXE-2 site abrogates the increase in transcriptional activity. Similar results were also seen when human *SOX10 *cDNA was used in co-transfection experiments in NIH3T3 cells (Additional file 2, Figure S2). On the other hand, *AP2 *was unable to significantly transactivate *ERBB3*_MCS6 in NIH3T3 cells (Additional file 2, Figure S2). Also, when added in combination with *SOX10*, it was unable to increase transcriptional activity of *ERBB3*_MCS6 in a manner that is more than additive when compared to *SOX10 *and *AP2 *independent transactivation, further suggesting that *AP2 *and *SOX10 *do not interact to regulate *ERBB3*_MCS6.

To further investigate the role of *Sox10 *in regulating *ERBB3*_MCS6, we next determined whether established disease causing mutations in *Sox10 *might in part mediate their regulation of *Erbb3 *via *ERBB3_*MCS6. To this end, we used a *Sox10 *mutant cDNA expression construct (c.928_929ins), hereby referred to as *Sox10-ΔSTP *to transactivate *ERBB3*_MCS6. This mutant was first discovered in the *Dom *mouse model of Hirschsrpung disease [20,21] and has a single nucleotide (Guanine) insertion at nucleotide 929 of the coding sequence, leading to a translational frameshift. The resulting protein possesses an intact DNA binding domain but 99 novel nucleotides replace the putative activation domain. The resulting protein inhibits the activity of wild-type *Sox10 *in a dominant negative fashion. Co-transfection of *ERBB3_*MCS6 with *Sox10-ΔSTP *does not result in *ERBB3_*MCS6 activity (Figure 2G). Additionally, ΔSTP also compromises the transactivation of *ERBB3_*MCS6 by wild-type *Sox10 *as seen in cells co-transfected with both the wild-type and the mutant forms of *Sox10*. This implies that *ERBB3_*MCS6 plays an important role in the regulation of *Erbb3 *by *Sox10*.

If *ERBB3_*MCS6 were indeed an important link in the regulation of *Erbb3 *expression by *Sox10*, we would expect the overexpression of wild-type *Sox10*, but not the *Sox10-ΔSTP *mutant, to increase endogenous transcript levels of *Erbb3*. In fact, when we examined the levels of *Erbb3 *transcript in Neuro2A cells upon *Sox10 *overexpression, we found that it was seven-fold higher than *Erbb3 *transcript levels in the absence of *Sox10 *(Figure 2H). Furthermore, *Sox10-ΔSTP *was unable to increase *Erbb3 *transcript levels and it also impeded the ability of the wild-type protein to increase *Erbb3 *transcript levels. An increase in total *Sox10 *transcript levels upon overexpression was verified via real-time PCR (Additional file 3, Figure S3). Therefore, this implies that *Sox10 *is sufficient for the transcriptional activation of *Erbb3*.

### Sox10 physically binds *ERBB3*_MCS6

We next used electrophoretic mobility shift assays to determine if *ERBB3_*MCS6 physically binds protein in a manner dependent on the SOXE-2 motif. We generated 50 bp probes that span the SOXE-2 binding site and labeled the probes with biotin. We then incubated the probes with nuclear extract derived from melan-a cells and subjected the reactions to gel electrophoresis. As seen in Additional file 4, Figure S4, a factor in the melan-a nuclear extract bound the probe and retarded its migration in the gel, causing a shift. This binding was successfully competed away by using increasing amounts of unlabeled probe. To determine whether this binding was specific to the SOXE-2 binding site, we next mutated the SOXE-2 binding site and incubated unlabeled mutant probe with nuclear extract. As is seen in lane 5 of Additional file 4, Figure S4, the mutant cold probe was unable to compete away the binding, thus indicating that the gel shift seen is specific to binding at the SOXE-2 binding site. In order to assay whether Sox10 directly binds to *ERBB3*_MCS6, we used chromatin immunoprecipitation followed by quantitative PCR using an antibody directed against Sox10 in S16 cells. As shown in Figure 2I, the rat ortholog of *ERBB3*_MCS6 is enriched in Sox10 immunoprecipitated samples but not in IgG immunoprecipitated samples. This binding is specific to Sox10 as knocking down Sox10 protein levels in S16 via siRNAs caused a loss of enrichment of *ERBB3*_MCS6 upon Sox10 immunoprecipitated in these cells. Collectively, this implies that Sox10 directly binds *ERBB3*_MCS6.

### *ERBB3*_MCS6 directs reporter expression in a neural crest specific manner reminiscent of *erbb3b *expression in zebrafish

**Figure 3:**
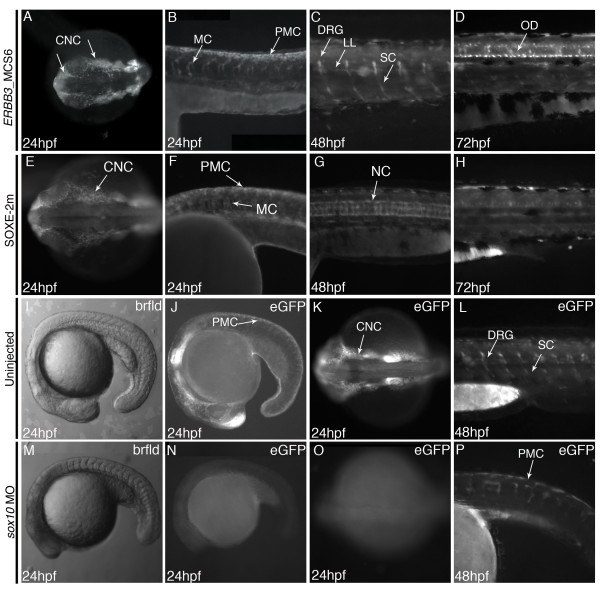
***ERBB3_*MCS6 drives reporter expression *in vivo *in a pattern similar to *erbb3b *and under the control of *sox10***. (A-D) Expression pattern of ERBB3_MCS driving eGFP in G1 transgenic 24-72hpf zebrafish embryos. Arrows indicate tissues where expression was noted in multiple founders. (E-H) eGFP expression pattern seen in multiple founders of SOXE-2m *ERBB3*_MCS6 transgenic fish. Although expression is similar to WT *ERBB3*_MCS6 at 24hpf, expression differs at later stages of development. (I-P) Results of morpholino knockdown of sox10 in *ERBB3*_MCS6 transgenic embryos. (I-L) Normal expression of ERBB3_MCS6 in uninjected transgenic embryos at 24-48hpf. (M-P) Loss of reporter expression in embryos injected with a Sox10 morpholino at 24hpf and appearance of disorganized GFP expressing NC cells at 48hpf. Abbreviations: cranial neural crest (CNC), migratory crest (MC), pre-migratory crest (PMC), dorsal root ganglia (DRG), posterior lateral line (PLL), Schwann cells (SC), oligodendrocytes (OD) and notochord (NC).

In order to confirm that *ERBB3*_MCS6 is an enhancer of *ERBB3 *in vivo, we generated stable transgenic zebrafish lines of *ERBB3*_MCS6 driving an eGFP reporter using a Tol2 based zebrafish transgenesis assay [39]. Briefly, we cloned *ERBB3*_MCS6 upstream of a *cfos *minimal promoter driving enhanced green fluorescent protein (eGFP). We then injected this sequence into 1-2 cell zebrafish embryos, screened G0 embryos for vector integration, and evaluated their offspring for germ line reporter expression. Reporter expression was assayed in four independent transgenic lines and compared to previously published patterns of endogenous *Erbb3*/*erbb3b *expression [10-12,14]. Consistent with its strong *in vitro *regulatory potential, *ERBB3*_MCS6 drove reporter expression in NC populations as early as 24 hours post fertilization (hpf) in transgenic zebrafish in a manner reminiscent of *erbb3b *expression (Figure 3A, B). Expression was noted in cranial neural crest, migratory and pre-migratory crest, all regions where *Erbb3/erbb3b *is known to be expressed (Figure 3A, B) [11]. At 48 hpf, expression was noted in the dorsal root ganglia, posterior lateral line ganglia and in Schwann cell precursors, consistent with known *Erbb3/erbb3b *expression (Figure 3C) [12,13]. Finally, at 72 hpf expression was noted in cells that are consistent in shape and position with mature oligodendrocytes (Figure 3D) [40]. Therefore, *ERBB3*_MCS6 drives reporter expression *in vivo *in neural crest cells and its derivative tissues in a manner consistent with *erbb3b *expression. Two other sequences, *ERBB3*_MCS1 and MCS4 also drove consistent expression in multiple founders in NC-derived populations consistent with *Erbb3*/*erbb3b *expression (Additional file 5, Figure S5). Although *ERBB3*_MCS1 did not show significant enhancer activity *in vitro*, or H3K4me1 marks, it demonstrated expression *in vivo *in a manner consistent with *Erbb3 *expression. This sequence directed reporter expression starting at 48 hpf in the mesencephalon, olfactory bulbs, cranial ganglia and pharyngeal arches, all regions of demonstrated *Erbb3 *expression [14,41]. *ERBB3*_MCS1 also directed expression in the midbrain-hindbrain boundary and in the hindbrain, regions where *Erbb3 *function has been shown to be essential [9] (Additional file 5, Figure S5A, B, C). Furthermore, this element directed expression in cells lining the anterior lateral line neurons and the epiphysis (data not shown). Consistent with its strong *in vitro *regulatory potential and H3K4me1 marks, *ERBB3*_MCS4 directed reporter expression *in vivo. ERBB3_*MCS4 drove eGFP expression *in vivo*, beginning at 24 hpf in the mesencephalon, the hindbrain and the pharyngeal arches. It also directed expression in the myotome consistent with endogenous *Erbb3 *expression [10,11] (Additional file 5, Figure S5D, E, F). This suggests that *ERBB3*_MCS1, MCS3 and MCS6 are all NC-directed enhancers of *ERBB3*.

### SOXE-2 motif is necessary for the activity of ERBB3_MCS6 *in vivo *in zebrafish

To determine the contribution of the SOXE-2 motif to the proper expression of *ERBB3*_MCS6 in vivo, we generated stable transgenic zebrafish lines with *ERBB3*_MCS6 harbouring a mutation in the SOXE-2 motif. We identified three independent stable transgenic zebrafish lines with the SOXE-2m *ERBB3*_MCS6 construct directing eGFP expression. eGFP expression was inconsistent among these lines. This may either be a direct result of position effect based on the position of integration of the vector, or mutation of the SOXE-2 motif may make the sequence more amenable to position effect due to loss of proper regulatory control. In one of the three lines, eGFP expression was not seen at 24 hpf whereas in the remaining two lines eGFP expression was qualitatively similar to expression directed by the WT *ERBB3*_MCS6 construct at 24 hpf (Figure 3E, F). Additionally, mutation of this site may yield quantitative differences in eGFP expression between the WT and mutant constructs. Technical reasons such as variation in transgene copy number and position effect preclude the use of this assay to evaluate such differences. Therefore, we cannot exclude the possibility that additional elements within *ERBB3*_MCS6 may be sufficient for its expression in NC in the early embryo. However, at 48 hpf and 72 hpf, expression driven by the mutant construct was qualitatively different across all founders as compared to expression directed by the WT construct. Expression was lost from dorsal root ganglia cells and Schwann cells in 48 hpf SOXE-2m *ERBB3*_MCS6 transgenic embryos. Expression domains were gained within these mutants in the notochord and cells of the ventral spinal cord (Figure 3G and data not shown). Furthermore, at 72 hpf expression was not seen in oligodendroglial cells in SOXE-2m *ERBB3*_MCS6 transgenic embryos (Figure 3H). However, at both 48 hpf and 72 hpf, expression in the ectomesenchymal derivatives of the NC such as in the pharyngeal arches was maintained in the mutant transgenic fish (data not shown). This is in agreement with a known role for *Sox10 *in non-ectomesenchymal but not in ectomesenchymal NC populations in the zebrafish [42]. This suggests that additional elements within *ERBB3*_MCS6 are sufficient for the expression of this sequence in ectomesenchymal derivatives of the NC. Therefore, the SOXE-2 motif is important for the proper expression of *ERBB3*_MCS6 in non-ectomesenchymal neural crest derivatives such as dorsal root ganglia and Schwann cells, but not in premigratory and migratory crest cells or ectomesenchymal NC derivatives. However, based on the diminution and not complete abrogation of *ERBB3*_MCS6 activity *in vitro*, we expect that mutation of this site may produce quantitative effects on the expression of *ERBB3*_MCS6, which cannot be assayed by zebrafish transgenesis. Therefore, to address the role of *Sox10 *in regulation *ERBB3*_MCS6 *in vivo*, we used morpholinos analysis in zebrafish.

### sox10 is necessary for the activity of *ERBB3*_MCS6 *in vivo *in zebrafish

In order to determine whether Sox10 is required for the enhancer activity of *ERBB3*_MCS6 *in vivo *similar to its requirement *in vitro*, we used morpholinos against *sox10 *in the stable transgenic *ERBB3*_MCS6 zebrafish to assay the effect of sox10 depletion on *ERBB3*_MCS6 activity. Using a previously published translation-blocking morpholino against the zebrafish *sox10 *gene product, we knocked down levels of *sox10 *in the *ERBB3*_MCS6 transgenic zebrafish [43]. Depletion of sox10 in zebrafish produces a phenocopy of the *colourless *(*sox10*^-/-) ^zebrafish mutant phenotype, which has been studied in detail [42]. Although NC cells form upon *sox10 *depletion, they accumulate in the premigratory position and fail to migrate and differentiate appropriately into their subsequent lineages [42,43]. Thus, if *sox10 *were necessary for *ERBB3*_MCS6 expression, we would expect to see a loss of *ERBB3*_MCS6 driven reporter expression in premigratory NC as well as a subsequent loss of expression in NC derived cell types such as Schwann cells and DRG due to the loss of these populations. Consistent with our expectation, upon knocking down *sox10*, we noted a complete loss of *ERBB3*_MCS6 activity in the developing NC in one-day old embryos (Figure 3N,O). This is in sharp contrast to the *ERBB3*_MCS6 driven reporter expression seen in the premigratory and migratory NC of uninjected embryos (Figure 3J,K). Since the sox10 morpholino produces embryos with a range of phenotypes, in embryos that show a weaker phenotype, we noticed a reduced number of eGFP expressing premigratory NC cells and highly reduced numbers of migrating crest at 24 hpf, consistent with a role for Sox10 in regulation *ERBB3*_MCS6 (Additional file 6, Figure S6C, D). eGFP expression in the cranial NC was affected to a lesser degree in these embryos (Additional file 6, Figure S6C). However, eGFP expression in 24 hpf embryos was noted in only 6/130 injected embryos (4.6%), which is in sharp contrast to the 27/60 uninjected (45%) embryos that showed eGFP expression at 24 hpf. By 48 hpf, *ERBB3*_MCS6 driven eGFP expression was noted in morpholino injected embryos in cells that are consistent in position with premigratory NC (Figure 3P). Expression was also noted in some migratory NC, however, these cells are highly disorganized as compared to the eGFP expressing cells seen in uninjected embryos consistent with migratory crest previously described for this model (Figure 3L, P) [42,43]. eGFP expression persists in these non-migrating NC cells upto 96hpf (data not shown) but is not seen in NC derived cells. Therefore, this suggests that sox10 is necessary for the timely and proper expression of *ERBB3*_MCS6 in neural crest cells and its derivative lineages *in vivo *in zebrafish.

## Discussion

Although *ERBB3 *is an important gene in the development and differentiation of a range of NC-derived populations, not much is known about the sequences and factors modulating its transcriptional output. *Sox10*, however, has been shown to influence *Erbb3 *levels [10], although, whether this regulation is direct or indirect was unknown. In this study, we identify novel regulatory enhancers of *ERBB3 *that direct expression in NC derived cell lines and exhibit epigenetic marks consistent with enhancer activity. One of these enhancers, *ERBB3*_MCS6 directs the strongest reporter expression *in vitro *and a broad overlap with endogenous *erbb3b *expression *in vivo *in zebrafish. We demonstrate that *ERBB3*_MCS6 is dependent upon and is responsive to Sox10 both *in vitro *and *in vivo*. Furthermore, we also demonstrate that Sox10 is both necessary and sufficient *in vitro *for endogenous *Erbb3 *expression. Finally, we demonstrate that Sox10 physically binds to *ERBB3*_MCS6, suggesting that *SOX10 *mediates its effect on *ERBB3*, at least in part, directly through *ERBB3*_MCS6.

Using a combination of sequence constraint, *in vitro *luciferase and chromatin immunoprecipitation assays, and *in vivo *zebrafish transgenesis, we have identified three functional enhancers of *ERBB3- ERBB3*_MCS1, MCS4 and MCS6. Although *ERBB3*_MCS1 did not significantly drive luciferase activity *in vitro *or exhibit enrichment of H3K4me1 marks, it drove reporter expression in transgenic zebrafish embryos in a manner consistent with endogenous *erbb3b *expression. Furthermore, although elements *ERBB3*_MCS2, MCS3 and MCS7 showed increased luciferase activity in vitro, they did not exhibit NC-directed enhancer activity *in vivo*. This suggests that although *in vitro *assays are useful in assaying the regulatory potential of enhancer sequences, they represent a single stage and cell type in development as compared to the full spectrum of NC derivatives that can be assayed *in vivo *and can potentially overlook regulatory sequences, which may be active at different stages or cell types or may attribute activity to sequences that are not biologically functional. Similarly, comparison of H3K4me1 marks and enhancer activity in the elements tested demonstrates that H3K4me1 is not sufficient to distinguish between active and inactive enhancers. *ERBB3*_MCS5, MCS7 and MCS8 showed very high levels of H3K4me1, but did not direct transcriptional activity *in vitro *or *in vivo*, suggesting that additional epigenetic or transcriptional marks may be necessary to distinguish them from active enhancers. These data are consistent with recent reports that suggest that additional epigenetic marks such as H3K27ac may be necessary to distinguish between H3Kem1 marked active and poised enhancers [28,29].

The intronic enhancer *ERBB3_*MCS6 demonstrated high transcriptional activity *in vitro *and also exhibited H3K4me1 marks. Furthermore, it directed expression almost exclusively, and broadly, in developing NC populations. The role of *AP2 *in regulating *ERBB3*_MCS6 remains unclear. Mutation of the AP2 binding site does not significantly reduce transcriptional activity of *ERBB3*_MCS6, suggesting that it is not required for the activity of *ERBB3*_MCS6. Furthermore, ectopic expression of *AP2 *does not increase transcriptional activity of *ERBB3*_MCS6 (Additional file 2, Figure S2). However, knockdown of *Ap2 *caused a significant decrease in transcriptional activity of *ERBB3*_MCS6 (Figure 2B). Therefore, although *AP2 *may be necessary for the activity of *ERBB3*_MCS6, it is not sufficient for transcriptional activity of *ERBB3*_MCS6, suggesting that it may act in concert with other transcription factors to mediate its effect on *ERBB3*_MCS6. Based on the results of our assays, it does not seem that *AP2 *interacts with *SOX10 *to regulate *ERBB3*_MCS6, although we cannot exclude the possibility that the contribution of *AP2 *to the regulation of *ERBB3*_MCS6 may be rate limited by and secondary to regulation by *SOX10*. Therefore, further examination will be required to determine how *AP2 *may regulate *ERBB3*_MCS6 and what, if any, co-factors may mediate this regulation. Since *AP2 *is known to regulate *ERBB3*, it is not surprising that we see a decrease in Erbb3 protein levels upon *Ap2 *knockdown in melan-a cells [35,36], however whether this decrease is mediated in part via *ERBB3*_MCS6 will require further investigation. Similarly, it will also be interesting to determine what, if any, other TFs may be involved in regulating *ERBB3 *via *ERBB3*_MCS6 by expanding the search for functionally important sequence motifs therein.

However, by focusing our investigation on the role of *SOX10 *in regulating *ERBB3*_MCS6, we show that it is directly responsive to *SOX10*. Although there were two potential *Sox10 *binding sites in *ERBB3_*MCS6, only one of them, SOXE-2 was required in our *in vitro *assays. This site is perfectly conserved across mammals and mutation of the site causes a loss of *Sox10 *responsiveness of *ERBB3_*MCS6. Furthermore, mutation of the SOXE-2 site affects the expression of *ERBB3*_MCS6 in non-ectomesenchymal NC derivatives in zebrafish, but not in ectomesenchymal derivatives of NC or in early premigratory and migratory NC. Although quantitative differences in expression of *ERBB3*_MCS6 due to the SOXE-2 mutation cannot be ruled out as they are below the threshold of detection by the zebrafish transgenesis assay, this data suggests that other motifs within *ERBB3*_MCS6 may be sufficient for its expression in early NC and ectomesenchymal derivatives. However, using additional *in vitro *and *in vivo *assays, we established an important role for *Sox10 *in regulating *ERBB3*_MCS6 via the SOXE-2 motif. Knockdown of *Sox10 *abrogates *ERBB3_*MCS6 activity, whereas ectopic expression of *Sox10 *induces *ERBB3_*MCS6 response, demonstrating that *Sox10 *is both necessary and sufficient for *ERBB3_*MCS6 transcriptional activity. Similarly, we also show that *Sox10 *is both necessary and sufficient for *Erbb3 *expression. This is consistent with previous data suggesting that *Erbb3 *is under the control of *Sox10 *[10]. Using gel-shift and chromatin immunoprecipitation assays, we also demonstrate direct binding of Sox10 to *ERBB3_*MCS6, most likely mediated through the SOXE-2 site. Finally, we use morpholino analysis in *ERBB3*_MCS6 transgenic zebrafish to demonstrate a requirement for Sox10 in the proper expression of this enhancer. Therefore, our data suggest that *ERBB3_*MCS6 may play a critical role in mediating the regulation of *ERBB3 *by *SOX10*.

## Conclusions

In conclusion, we have identified three transcriptional enhancers at the *ERBB3 *locus, whose expression domains overlap NC-derived populations. We show that one such enhancer (*ERBB3_*MCS6) directs reporter expression broadly in NC cells and, like *ERBB3*, is directly responsive to *Sox10*, implying that *Sox10 *may act through this enhancer to regulate *ERBB3 *transcription in the NC.

## Methods

### Fish husbandry and transgene and morpholino injections

Zebrafish were bred and maintained as previously described [44,45]. Microinjections were carried out as previously described [39]. Briefly, eGFP expression vectors were injected into 1-2 cell stage embryos (n≥300). Reporter expression was assayed between 24hpf-5dpf and embryos with consistent GFP expression were selected and raised to adulthood and founders were identified. For morhpholino experiments, previously published sox10 morpholino was ordered from Gene Tools (Philomath, OR) [43]. 6.6 ng of the morpholino was injected into each embryo by microinjection. Embryos were analyzed and imaged using a Carl Zeiss Lumar V12 Stereo microscope with AxioVision version 4.8 software. Transgenic lines for *ERBB3*_MCS1, *ERBB3*_MCS4 and *ERBB3*_MCS3 are listed at the Zebrafish International Resource Center (ZIRC) (http://zebrafish.org) under allele designations JH112, JH113 and JH114 respectively.

### Identification of conserved non-coding sequences and transcription factor binding sites

Conserved sequences were identified at the human *ERBB3 *locus and upstream and downstream intergenic regions (chr12:54,724,274-54,784,370) using the PhastCons custom track on the UCSC Genome Browser (http://genome.ucsc.edu) on genome build hg18. We used the 17-way MutiZ alignment to identify the most conserved sequences within the introns of the gene and within intergenic regions surrounding the gene. The coordinates of the MCSs and the primers used to amplify them are shown in Additional file 7, Table S1.

Fasta format DNA sequence of *ERBB3_*MCS6 from the genome browser was used to query MatInspector [46], MATCH 1.0 and Jaspar databases for identifiable TFBS using default settings.

### Vector construction and mutagenesis

Expression vectors were constructed using Gateway Technology (Invitrogen, Carlsbad, CA). The desired genomic regions were amplified by with attB-flanked primers and recombined into the pDONR221 vector. Successful recombination was confirmed by sequencing. Next, entry clones were recombined into the destination vectors pLGF-E1b for luciferase assays and pT2cfosGW for zebrafish injections [25,47].

*ERBB3_*MCS6 was mutated using the QuikChange II XL Site-directed mutagenesis kit (Stratagene, La Jolla, CA). Mutagenesis primers were designed to change the potential transcription factor binding sites (TFBS) to Hpa1 restriction sites using the QuikChange Primer Design tool. Primers are included in Additional file 8, Table S2.

*Sox10*-pcDNA3.1 and *Sox10-ΔSTP*-pcDNA3.1 were cloned using *Sox10*-pCMV and *Sox10-ΔSTP*-pCMV as templates. Coding sequence for human SOX10 and AP2 for transactivation experiments was amplified by PCR from I.M.A.G.E clone MGC-3510 and MGC-22117 respectively and cloned into pcDNA3.1 (Invitrogen, Carlsbad, CA) using In-Fusion PCR Advantage Cloning Kit (Clontech, Mountain View, CA). The PCR primers used were designed using In-Fusion Primer Design tool and are shown in Additional file 9, Table S3. Successful cloning was verified by sequencing.

The E189X *Sox10 *cDNA cloned into a pCMV promoter was a kind gift from Ken Inoue, Jim Lupski and Michael Wegner [23,38].

### Cell culture and transfection

Immortalized melanocytes (melan-a) and immortalized Schwann cells were maintained as described [48,49]. NIH 3T3 cells were grown in 10% FCS in DMEM under standard conditions. Neuro2A cells were grown in 10%FCS in MEM under standard conditions.

### Luciferase assay

melan-a cells were plated in 24-well plates 24 hours prior to transfection at a density of 4 × 10^4 cells/well. 400 ng of the luciferase reporter plasmids were cotransfected with 8 ng of CMV-RL renilla expression vector (Promega, Madison, WI). 48 hours after transfection, cell lysate was collected and assayed using the Dual-Luciferase Reporter Assay System (Promega, Madison, WI). For siRNA knockdown, 200 ng of luciferase reporter vectors were cotransfected with 5 pmol of each siRNA pool to a total of 10 pmol/well of a 24-well plate. Where appropriate, scrambled siRNA was added to maintain a final concentration of 10 pmol/well of siRNA.

Neuro2A cells were plated in 24-well plates 24 hours prior to transfection at a density of 5 × 10^4 cells/well. 400 ng of luciferase reporter plasmids were cotransfected with 200 ng of *Sox10*-pcDNA3.1 and *ΔSTP-Sox10*-pcDNA3.1. Where appropriate, empty pcDNA3.1 vector was added to maintain a final concentration of 800 ng/well of DNA. 8 ng of CMV-RL were added to each well. Cell lysate was collected 24 hours after transfection and assayed as mentioned above. Luciferase assays were carried out using a Tecan GENiosPro machine. All assays were performed in triplicate and repeated in at least two independent experiments.

For luciferase assays in S16 cells, 1 × 10^4 cells were plated in 96-well plates 24 hours prior to transfection with luciferase vectors. Each transfection reaction included 200 ng of experimental and control luciferase expression constructs and 2 ng of a renilla expression construct to control for transfection efficiency and cell viability. Cells lysates were collected 48 hours after transfection and luciferase assays were carried out with the Dual-Luciferase Assay System (Promega, Madison, WI) and analyzed on a Glomax Multi-Detection System (Promega, Madison, WI).

### siRNA knockdown

ON-TARGET*plus *SMARTpool siRNA was ordered against mouse *Sox10 *and *Tfap2a *from Dharmacon (Lafayette, CO). Knockdown was achieved by using 5 pmol of each siRNA in a 24-well transfection format.

### Western blot

Cells were trypsinized and washed twice in 1XPBS and resuspended in 2X Incomplete Lamelli buffer and passed through a QiaShredder (Invitrogen, Carlsbad, CA) to obtain whole cell lysate. Protein was quantified and run on a 10% Mini Protean TGX gel (Biorad, Hercules, CA), transferred onto a nitrocellulose membrane and blocked overnight in 5% non-fat dry milk block. Sox10 antibody was used at a dilution of 1.5 ng/ul (MAB2864, R&D Biosystems, Minneapolis, MN), AP2 antibody was used at 1:500 (ab52222, Abcam, Cambrigde, MA), ErbB3 antibody was used at 1:500 (sc285, Santa Cruz Biotechnology, Santa Cruz, CA) and anti-tubulin at 1:3000 (CP06, Calbiochem, San Diego, CA). HRP-conjugated secondary was used and antibody binding was visualized using SuperSignal West Dura Extended Duration Substrate (34076, Thermo Fischer, Rockford, IL). Membranes were stripped using Restore PLUS Western Blot Stripping Buffer (46430, Thermo Fischer, Rockford, IL

### RNA extraction, cDNA synthesis and Real-time PCR

Neuro2A cells were plated in 6-well dish at a density of 2.5 × 10^5 cells/well using Lipofectamine 2000 (Invitrogen, Carlsbad, CA). A total of 2 ng of *Sox10*-pcDNA3.1 and *ΔSTP-Sox10*-pcDNA3.1 were transfected into the cells and RNA was collected 24 hours later using the RNeasy Mini Kit (Qiagen, Valencia, CA) using manufacturers instructions. Subsequently, cDNA was synthesized using the SuperScriptIII First Strand Synthesis System for RT-PCR (Invitrogen, Carlsbad, CA). Real-time PCR was then performed in triplicate using the Universal Gene Expression Master Mix (ABI, Carlsbad, CA) and the PrimeTime qPCR Assay designed against mouse *Sox10 *and *ErbB3*. Real-time PCR and analysis were performed on the Opticon2 (Biorad, Hercules, CA).

### Electrophoretic mobility shift assay

Probes spanning the SOXE-2 binding site were designed (Additional file 10, Table S4) and labeled with the Biotin 3' End DNA Labeling Kit (Pierce, Rockford, IL) according to manufacturers instructions. Nuclear extract was made from melan-a cells using the NE-PER Nuclear and Cytoplasmic Extraction Reagents (Pierce, Rockford, IL). EMSAs were performed using the LightShift Chemiluminescent EMSA Kit (Pierce, Rockford, IL). Briefly, 12.5 fmol of labeled probe was incubated with nuclear extract in the presence of binding buffer and 1 ug of poly (dI.dC) in a 20 ul reaction for 10 min at room temperature. For competition assays, 500 and 1000 molar fold excess of unlabeled probe was added. Products were run on precast polyacrylamide gels (4-20% or 7.5%) (Biorad, Hercules, CA) and signal was developed using the LightShift Chemiluminescent kit.

### Chromatin Immunoprecipitation (ChIP) and Real-time PCR

ChIP was performed in melan-a cells as previously described [50] with some changes. Each ChIP experiment was performed with ~1 × 10^8 ^cells. Alternative lysis buffers to those in the referenced protocol were used, as follows: Lysis buffer 1 (5 mM PIPES, 85 mM KCl, 0.5% NP-40, and 1 × Roche Complete, EDTA-free protease inhibitor), lysis buffer 2 (50 mM Tris-HCl, 10 mM EDTA, 1% SDS, and 1 × Roche Complete, EDTA-free protease inhibitor), and lysis buffer 3 (16.7 mM Tris-HCl, 1.2 mM EDTA, 167 mM NaCl, 0.01% SDS, 1.1% Triton X-100, and 1 × Roche Complete, EDTA-free protease inhibitor). Sonication was performed with a Bioruptor (Diagenode, Denville, NJ) with the following settings: high output; 30 second disruption; 30 second cooling; total sonication time of 35 min with addition of fresh ice and cold water to water bath every 10 minutes. 2 ug of antibody specific to H3K4me1 (ab8895, Abcam Cambridge, MA) and non-specific IgG (ab46540, Abcam Cambridge, MA) was used for immunoprecipitation. IP wash conditions were also adjusted from the above referenced protocol, as follows: Each IP was washed twice with low salt wash buffer (0.1% SDS, 1% Triton X-100, 2 mM EDTA, 20 mM Tris-HCl, 150 mM NaCl), twice with high salt wash buffer (0.1% SDS, 1% Triton X-100, 2 mM EDTA, 20 mM Tris-HCl, 500 mM NaCl), twice with cold LiCl wash buffer (0.25M LiCl, 1% IGEPAL CA630, 1% deoxycholic acid (sodium salt), 1 mM EDTA, 10 mM Tris-HCl), and rinsed once with PBS, pH 7.4. Immunoprecipitated DNA was analyzed by real-time PCR using SYBR Green (ABI, Carlsbad, CA) and primers shown in Additional file 11, Table S4 and performed and analyzed on an Opticon2 (Biorad, Hercules, CA) using the % Input method. Primer sequences for PCR are given in supplementary table 5.

ChIP in S16 cells was performed as previously described [51]. The antibodies used were anti-Sox10 (sc-17342X, Santa Cruz Biotechnology, Santa Cruz, CA) and control anti-goat IgG (sc-2808, Santa Cruz Biotechnology, Santa Cruz, CA). ChIP was analyzed in duplicate by quantitative PCR and analyzed by the % Input method. Primers used for PCR are shown in Additional file 11, Table S4.

## Competing interests

The authors declare that they have no competing interests.

## Authors' contributions

MKP, SKL, WJP and ASM conceived of the study. MKP designed and performed the experiments, analyzed all data, and wrote the manuscript. XR and DG performed the ChIP experiment, JC participated in the luciferase assays, SKL contributed to the study design and data analysis, identified the MCS elements and cloned them into pe1B and pXIG vectors, ARM, KC and SJ participated in the creation and analysis of zebrafish transgenics, EAJ and JS did the chromatin immunoprecipitation assays in S16 cells, CJH and AA did the luciferase assays in S16 cells, WJP participated in the design and analysis of the study, ASM participated in the design and analysis of the study and helped write the manuscript. All authors read and approved the final manuscript.

## Supplementary Material

Additional file 1 **Figure S1 - Identification of putative neural crest transcription factor binding sites in *ERBB3_*MCS6**. Sequence of intronic neural crest enhancer *ERBB3_*MCS6 showing the location of putative transcription factor binding sites (TFBS) identified using multiple TFBS search programs. SOXE-2 adheres to the SOXE binding consensus sequence. Click here for file

Additional file 2 **Figure S2 - Transcriptional transactivation of *ERBB3_*MCS6 by SOX10 and AP2**. Luciferase assay of wild type *ERBB3_*MCS6 in NIH3T3 cells when transiently co-transfected with equal amounts of an empty expression vector (pcDNA.31), *SOX10 *cDNA or *AP2 *cDNA. Cell lysates were collected 24 hours post transfection. All values are normalized to a renilla internal control and shown as fold-change compared to the promoter only construct (pe1B) with standard deviation. Click here for file

Additional file 3 **Figure S3 - Increase in Sox10 transcript levels upon Sox10 overexpression**. Real-time PCR results showing an increase in WT and mutant *Sox10 *cDNA upon transient transfection of WT and *Sox10-ΔSTP *cDNA in Neuro2A cells. Values are normalized to an 18S internal control and shown as a fold-change compared to the promoter only construct (pcDNA3.1) with standard error. Click here for file

Additional file 4 **Figure S4 - A nuclear protein within melan-a cells binds SOXE2**. EMSA demonstrating binding of *ERBB3_*MCS6 to a protein in melan-a nuclei at the SOXE-2 site. Nuclear extract binds free probe (Lane 1) and shifts it upwards (Lane 2). Addition of 500X (Lane 3) and 1000X (Lane 4) molar excess of unlabeled probes competes shift. Addition of cold unlabeled probe with a mutation in the SOXE-2 binding site does not compete away the shift (Lane 5). Click here for file

Additional file 5 **Figure S5 - *ERBB3_*MCS1 and *ERBB3_*MCS4 drive reporter expression *in vivo *in a pattern similar to *erbb3b***. (A-J) Expression pattern of the indicated MCS driving eGFP in G1 transgenic 24-72hpf zebrafish embryos. Arrows indicate tissues where expression was noted in multiple founders. Abbreviations: mesencephalon (M), hindbrain (HB), olfactory bulb (OB), pharyngeal arches (PA), cranial ganglia, posterior lateral line ganglia (PLLg). Click here for file

Additional file 6 **Figure S6- Range of eGFP phenotypes in *ERBB3*_MCS6 transgenic fish upon sox10 morpholino injection**. (A-B) eGFP expression driven by *ERBB3*_MCS6 in uninjected fish at 24hpf. Expression is noted in cranial neural crest (CNC), premigratory NC (PMC) and migratory crest (MC) (C-D) Fewer eGFP positive CNC (C) and PMC cells seen in sox10 morpholino injected transgenic embryos, and significantly reduced numbers of MC (D). Click here for file

Additional file 7 **Table S1- Coordinates of the *ERBB3*_MCS8 elements (human genome build hg18) and primers used for PCR amplification of each element**. Click here for file

Additional file 8 **Table S2- Primers used for site-directed mutagenesis of *ERBB3*_MCS6**. Click here for file

Additional file 9 **Table S3- Primers used for cloning WT and mutant *sox10 *cDNA, *AP2 *and *SOX10 *cDNA**. Click here for file

Additional file 10 **Table S4- Probes used for EMSA assay**. Click here for file

Additional file 11 **Table S5- Primers used for qPCR analysis of ChIP assay**. Click here for file
