# Supplementary figures and images for: SOX10 directly modulates ERBB3 transcription via an intronic neural crest enhancer
Source: BMC Dev Biol. 2011 Jun 14;11:40. doi: 10.1186/1471-213X-11-40 (PMC3124416; doi:10.1186/1471-213X-11-40)

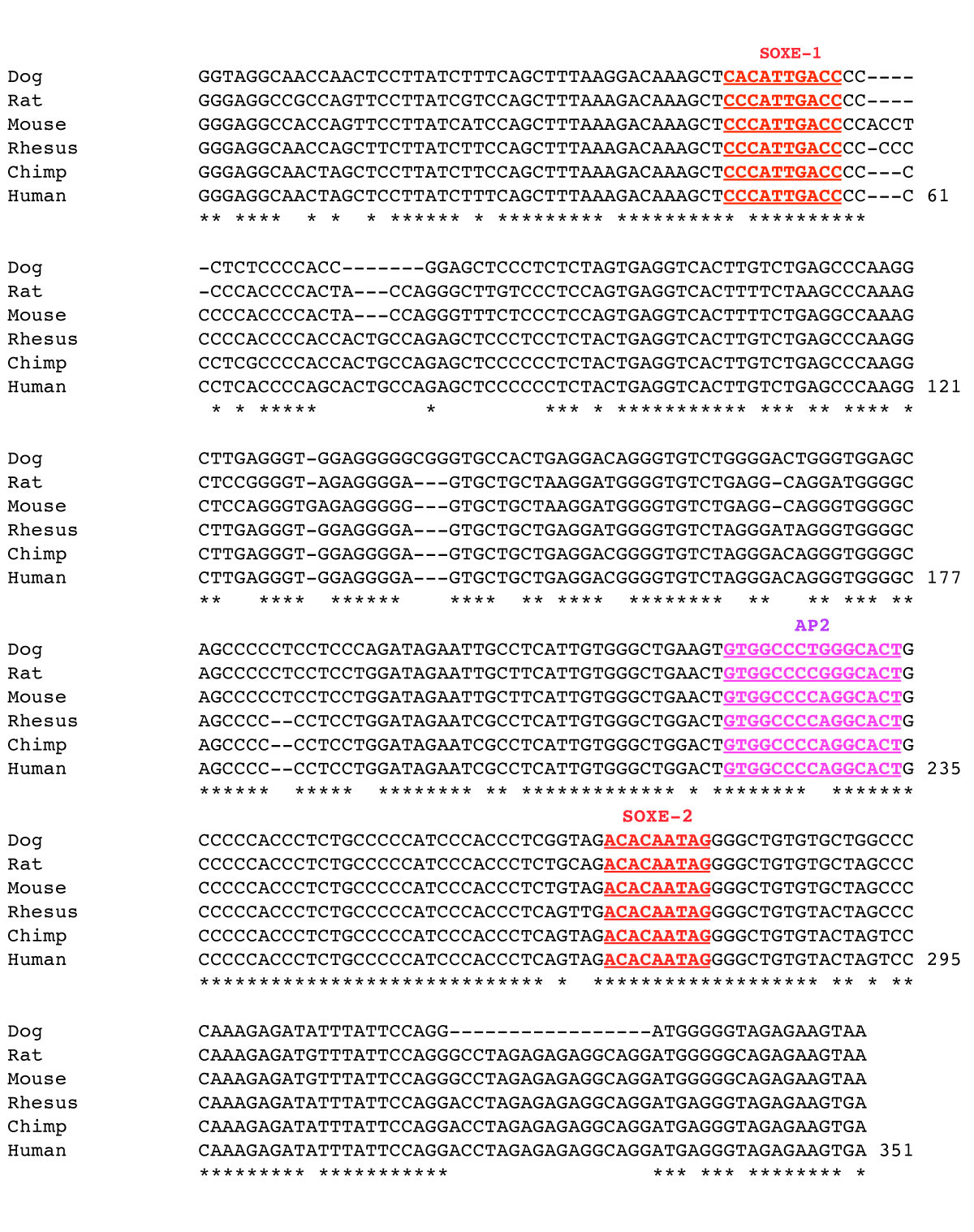

Supplement: Additional file 1 — Figure S1 - Identification of putative neural crest transcription factor binding sites in ERBB3_MCS6. Sequence of intronic neural crest enhancer ERBB3_MCS6 showing the location of putative transcription factor binding sites (TFBS) identified using multiple TFBS search programs. SOXE-2 adheres to the SOXE binding consensus sequence. [file 1471-213X-11-40-S1.TIFF]

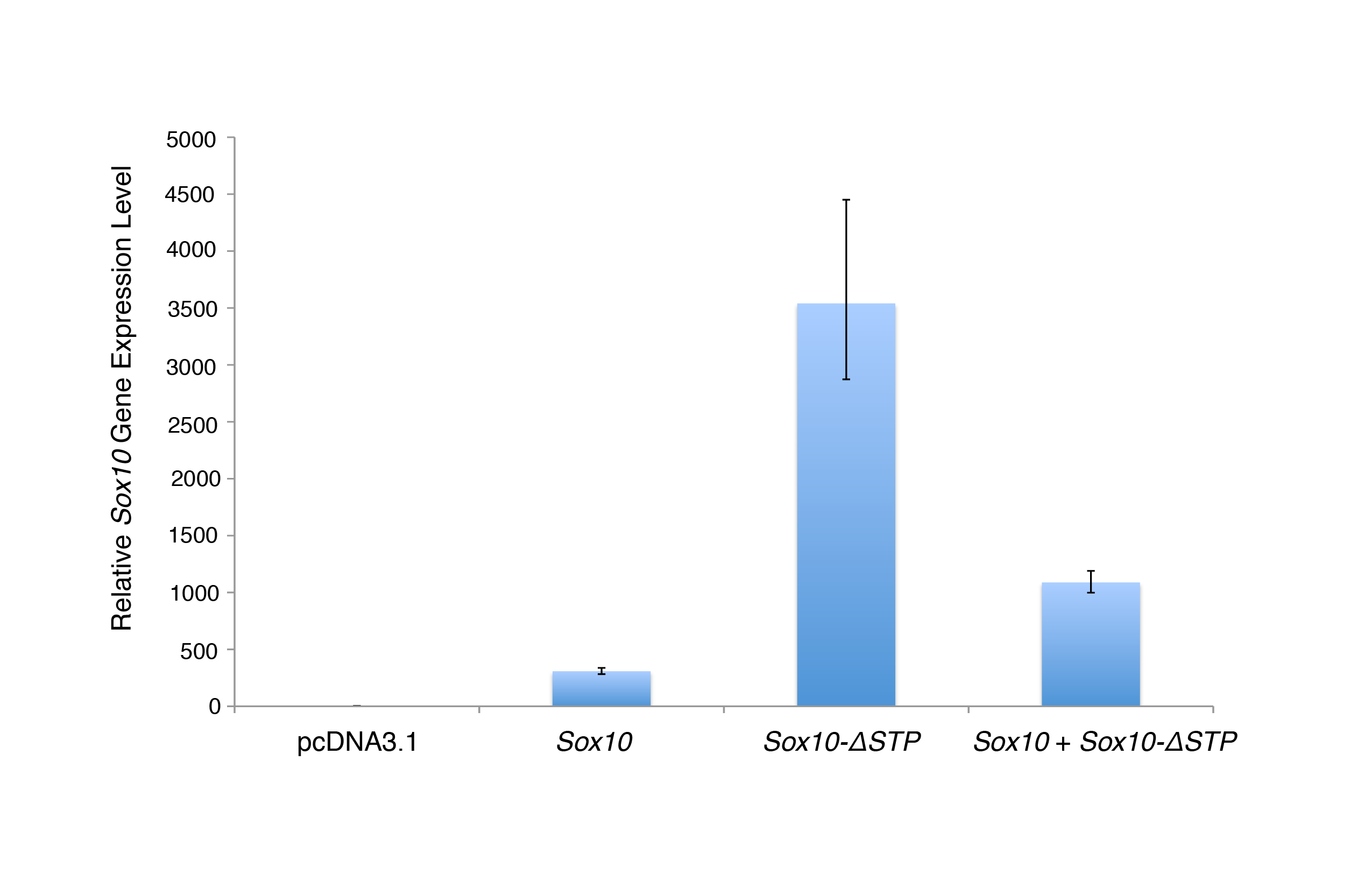

Supplement: Additional file 3 — Figure S3 - Increase in Sox10 transcript levels upon Sox10 overexpression. Real-time PCR results showing an increase in WT and mutant Sox10 cDNA upon transient transfection of WT and Sox10-ΔSTP cDNA in Neuro2A cells. Values are normalized to an 18S internal control and shown as a fold-change compared to the promoter only construct (pcDNA3.1) with standard error. [file 1471-213X-11-40-S3.TIFF]

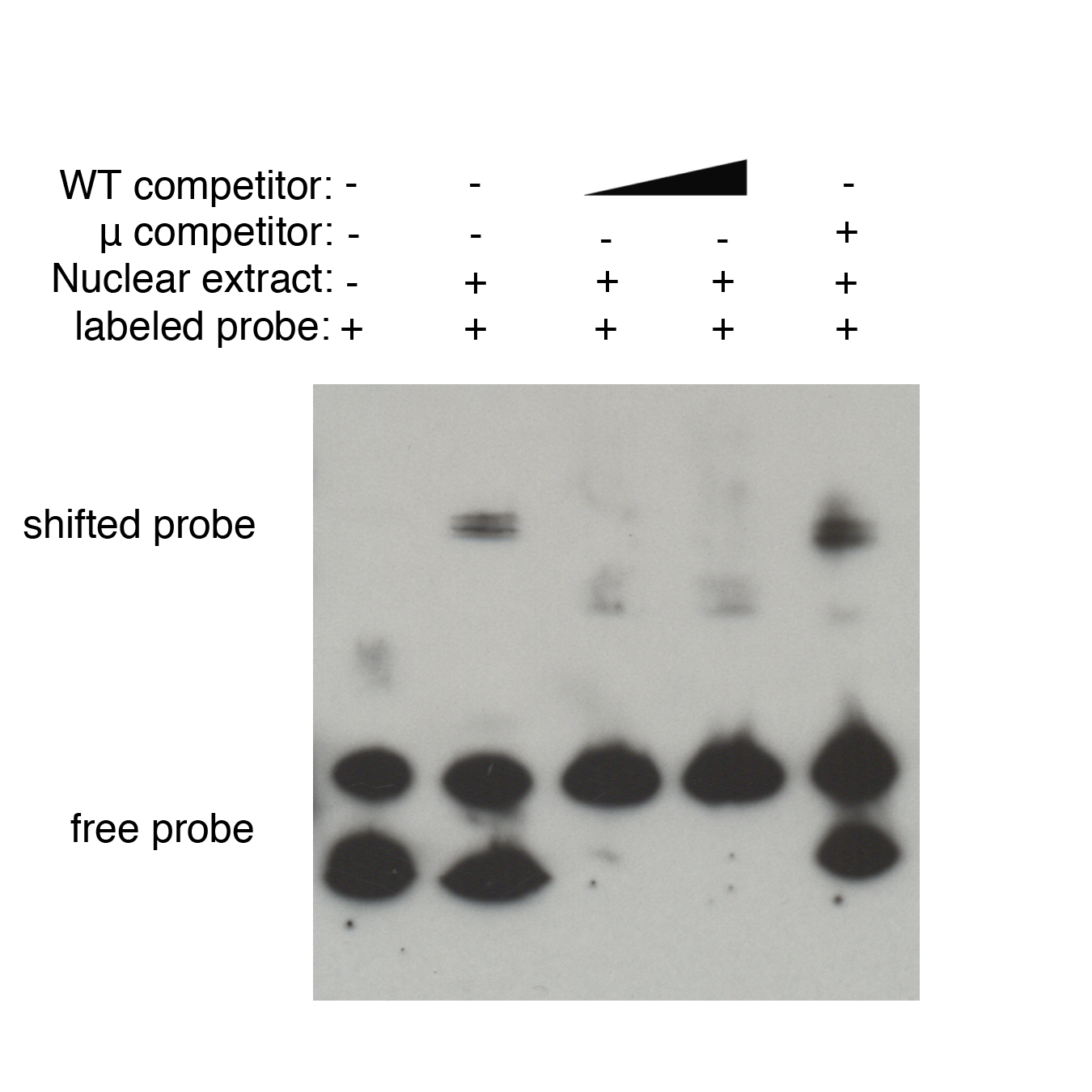

Supplement: Additional file 4 — Figure S4 - A nuclear protein within melan-a cells binds SOXE2. EMSA demonstrating binding of ERBB3_MCS6 to a protein in melan-a nuclei at the SOXE-2 site. Nuclear extract binds free probe (Lane 1) and shifts it upwards (Lane 2). Addition of 500X (Lane 3) and 1000X (Lane 4) molar excess of unlabeled probes competes shift. Addition of cold unlabeled probe with a mutation in the SOXE-2 binding site does not compete away the shift (Lane 5). [file 1471-213X-11-40-S4.TIFF]

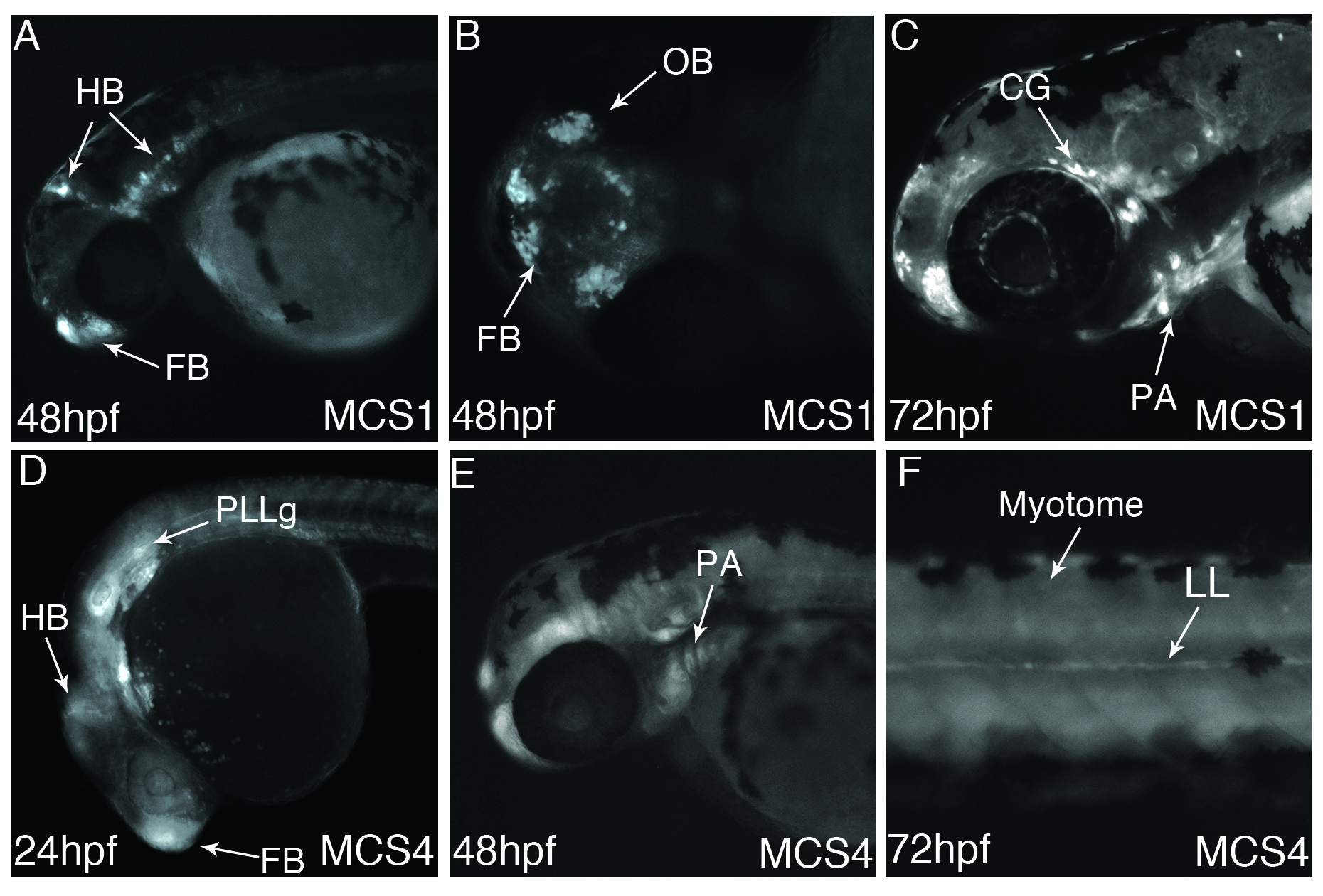

Supplement: Additional file 5 — Figure S5 - ERBB3_MCS1 and ERBB3_MCS4 drive reporter expression in vivo in a pattern similar to erbb3b. (A-J) Expression pattern of the indicated MCS driving eGFP in G1 transgenic 24-72hpf zebrafish embryos. Arrows indicate tissues where expression was noted in multiple founders. Abbreviations: mesencephalon (M), hindbrain (HB), olfactory bulb (OB), pharyngeal arches (PA), cranial ganglia, posterior lateral line ganglia (PLLg). [file 1471-213X-11-40-S5.TIFF]

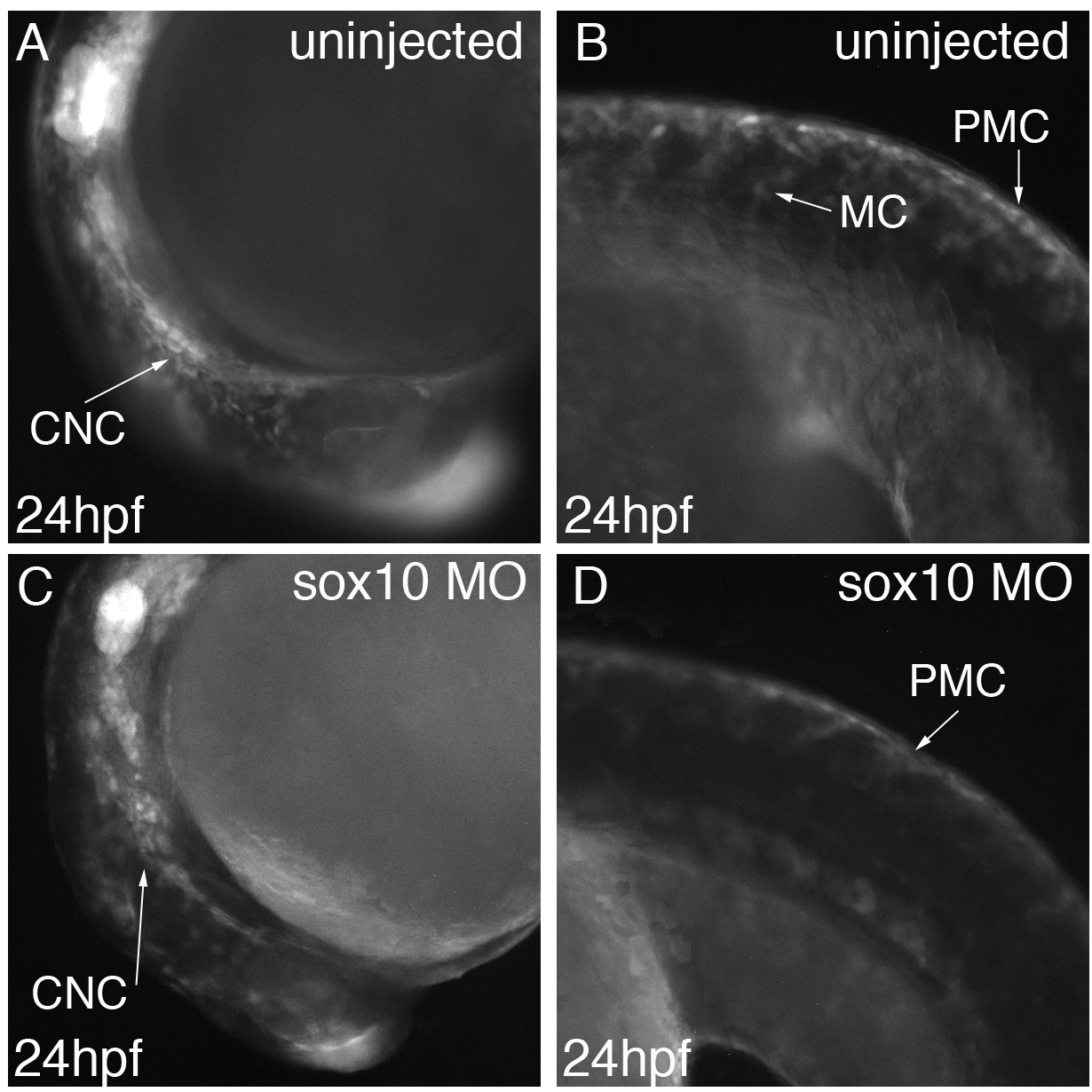

Supplement: Additional file 6 — Figure S6- Range of eGFP phenotypes in ERBB3_MCS6 transgenic fish upon sox10 morpholino injection. (A-B) eGFP expression driven by ERBB3_MCS6 in uninjected fish at 24hpf. Expression is noted in cranial neural crest (CNC), premigratory NC (PMC) and migratory crest (MC) (C-D) Fewer eGFP positive CNC (C) and PMC cells seen in sox10 morpholino injected transgenic embryos, and significantly reduced numbers of MC (D). [file 1471-213X-11-40-S6.TIFF]
